# Motor imagery as an intervention to improve activities of daily living post-stroke: A systematic review of randomized controlled trials

**DOI:** 10.1177/03080226221145441

**Published:** 2023-02-07

**Authors:** Kathryn JM Lambert, Cole Hoar, Jordan Houle, Catrin Motley, Natalie Ball, Ada WS Leung

**Affiliations:** 1 Department of Occupational Therapy, Faculty of Rehabilitation Medicine, University of Alberta, Edmonton, AB, Canada; 2 Neuroscience and Mental Health Institute, University of Alberta, Edmonton, AB, Canada

**Keywords:** Stroke, motor imagery, activities of daily living, randomized controlled trials, systematic review

## Abstract

**Introduction:**

Motor imagery (MI) may be an effective tool for improving activities of daily living (ADL) post-stroke. However, no review to date has examined ADL independence when investigating training effectiveness. This review aimed to evaluate the quality of evidence and the effectiveness of MI training for improving ADL independence post-stroke.

**Method:**

Randomized controlled trial (RCT) studies comparing MI to conventional therapies were reviewed. Methodological quality was assessed using the Physiotherapy Evidence Database (PEDro) scale.

**Results:**

Thirteen articles met inclusion criteria. The overall quality was considered moderate to good, with a PEDro score ranging from 3 to 8. Most studies (9 out of 13) were considered good quality, with one rating of poor quality and three of fair quality. The primary findings suggest that MI training is a low-risk tool that may facilitate ADL independence. Audio-based MI training seems to improve ADL independence when paired with other rehabilitation methods, but the results should be interpreted with caution.

**Conclusion:**

To our knowledge, this is the first systematic review to examine RCTs investigating MI effectiveness in improving ADL post-stroke. Results support the use of MI to facilitate ADL independence. However, more research is needed to establish practice guidelines for implementing MI training post-stroke.

## Introduction

Around half of stroke survivors experience long-term impairments in their ability to perform activities of daily living (ADL) (Miller et al., 2010). ADL fall into two categories: basic activities of daily living (BADL) and instrumental activities of daily living (IADL). BADL refer to the fundamental skills needed to manage one’s physical needs, such as toileting, eating, bathing, and mobilizing within one’s environment. BADL are often assessed using the Barthel Index (BI) and the Functional Independence Measure (FIM) (Schepers et al., 2006). Assessment scores capture the extent of assistance needed by the patients to complete different tasks, providing a measure of ADL independence. IADL are a set of more complex skills needed to live independently, such as cleaning, cooking, and money management, which are usually measured by performance tests (e.g., Liu et al., 2009).

Occupational therapy is essential to facilitating ADL independence following stroke (Steultjens et al., 2003). Treatment from an occupational therapist may include the training of lost performance skills or the teaching and practice of strategies that compensate for the stroke survivor’s new functional baseline (Steultjens et al., 2003). Both strategies enable patients to complete ADL as safely and independently as possible. Occupational therapy often involves task-oriented training, or the repeated physical practice of the target task to improve its performance (Steultjens et al., 2003). However, several factors can limit intensive physical task practice, including patient symptoms of pain, fatigue, or severe hemiplegia and systemic constraints that restrict dedicated therapy time (Luker et al., 2015).

One consideration is supplementing physical task repetitions with motor imagery (MI) or the mental rehearsal of movement without its physical performance. Regular use of MI facilitates motor skill acquisition in healthy and athletic populations (Ruffino et al., 2017). This facilitation is particularly significant when MI is paired with physical task practice (Ruffino et al., 2017). Brain imaging studies provide a possible explanation for these benefits, with MI training shown to induce both short- and long-term reorganization of sensorimotor cortices (Ruffino et al., 2017). This restructuring is proposed to facilitate other processes that rely on these networks, such as motor execution (Ruffino et al., 2017).

It has been demonstrated that MI training paired with physical practice is more effective at improving upper extremity function post-stroke than physical practice alone (Barclay et al., 2020). The effect of MI training on ADL independence is comparatively unclear. In two recent review studies, Guerra et al. (2017) and Barclay et al. (2020) reported that MI training may not improve ADL independence post-stroke. However, the results were based on a small sample of studies (e.g., 3 out of 32 studies in Guerra et al., 2017; 4 out of 25 studies in Barclay et al., 2020). High study heterogeneity and risk of bias also limited confidence in the documented null results (Barclay et al., 2020; Guerra et al., 2017).

Furthermore, for MI to be effectively implemented in stroke rehabilitation, one factor to consider is the instruction mode of MI training (Schuster et al., 2011). Patients may receive MI training through direct therapist instruction during in-person treatment sessions. Training can also be completed outside of direct therapist supervision through pre-recorded audio or visual-based instructions. This approach enables patients to participate in low-risk activities outside of time-restricted treatment sessions. The majority of MI training interventions in the rehabilitation literature have been delivered via pre-recorded instructions (Schuster et al., 2011). How different modes of instruction impact MI effectiveness on ADL independence has never been systematically reviewed.

To the best of our knowledge, no review has used ADL independence as its primary outcome measure when investigating the effectiveness of MI training in stroke rehabilitation. Given occupational therapy’s established role in facilitating ADL independence, an investigation into treatments that may reduce patient dependency on ADL is highly relevant to the profession (Steultjens et al., 2003). We thus carried out a systematic review with two objectives (1) to examine the quality of evidence investigating the effect of MI training on ADL independence post-stroke and (2) to evaluate the effectiveness of MI training on ADL independence post-stroke, with modes of MI instruction taken into consideration.

## Methods

### Search strategy

This systematic review was carried out in April 2021 and followed the guidelines recommended in the Preferred Reporting Items for Systematic Reviews and Meta-Analyses (PRISMA) statement (Moher et al., 2009). The search strategy was developed in collaboration with a health sciences librarian. Searches were conducted in CINAHL, Cochrane Library, PsycINFO, PubMed, and Scopus databases (see Table 1 for search strategy details). While quality of life was included in the initial search as a potential outcome of interest, it was later determined that the review should focus solely on independence in ADL as opposed to combining both outcomes, which are conceptually distinct. No limits such as English language, specific publication dates, region, or study design were applied to the search as reviewers wanted to safeguard against incidentally omitting any mis-indexed or yet-to-be-indexed articles. Search results were exported to Covidence (https://www.covidence.org), and duplicates were removed before being screened for eligibility.

**Table 1.** Line-by-line search strategies on the five databases.

CINAHL Plus With Full Text (EBSCO Interface)	S1: ((MH stroke) OR (stroke* OR “cerebrovascular accident” OR “ischemic stroke” OR poststroke)) S2: ((MH “Guided Imagery”) OR (“mental imag*” OR “kinesthetic imag*” OR “motor imag*” OR “visual imag*” OR “mental practic*” OR “mental training” OR “mental rehearsal” OR visualiz* OR visualis* OR “internal verbali*” OR “sensory imag*”)) S3: ((MH “Quality of Life+”) OR (fim FIM barthel OR ADL OR “activities of daily living” OR “daily life tasks” OR “activities of daily life” OR “quality of life” OR “patient satisfaction”)) S4: S1 AND S2 AND S3
Cochrane Library Search Strategy	1. MeSH descriptor: [Stroke] explode all trees 2. MeSH descriptor: [Imagery, Psychotherapy] explode all trees 3. MeSH descriptor: [Quality of Life] explode all trees 4. (stroke* or “cerebrovascular accident” or “ischemic stroke” or poststroke) 5. (“mental imagery” or “kinesthetic imagery” or “motor imagery” or “visual imagery” or “mental practice” or “mental training” or “mental rehearsal” or visualiz* or visualis* or “internal verbali*” or “sensory imag*”) 6. (fim or barthel or ADL or “activities of daily living” or “daily life tasks” or “activities of daily life” or “quality of life” or “patient satisfaction”) 7. #1 OR #4 8. #2 OR #5 9. #3 OR #6 10. #7 AND #8 AND #9
PsycINFO (OVID Interface)	1. (stroke* or “cerebrovascular accident” or “ischemic stroke” or poststroke).mp. 2. exp Guided Imagery/ 3. (“mental imag*” or “kinesthetic imag*” or “motor imag*” or “visual imag*” or “mental practic*” or “mental training” or “mental rehearsal” or visualiz* or visualis* or “internal verbali*” or “sensory imag*”).mp. 4. exp “Quality of Life”/ 5. (fim or barthel or ADL or “activities of daily living” or “daily life tasks” or “activities of daily life” or “quality of life” or “patient satisfaction”).mp. 6. 2 or 3 7. 4 or 5 8. 1 and 6 and 7
PubMed Search Strategy	((stroke OR “cerebrovascular accident” OR “ischemic stroke” or poststroke) AND (“mental imagery” OR “kinesthetic imagery” OR “motor imagery” OR “visual imagery” OR “mental practice” OR “mental training” OR “mental rehearsal” OR visualiz* OR visualis* OR “internal verbali*” or “sensory imag*”) AND (fim or barthel or ADL or “activities of daily living” or “daily life tasks” or “activities of daily life” or “quality of life” or “patient satisfaction”))
Scopus Search Strategy (Scopus Interface)	(TITLE-ABS-KEY (stroke OR “cerebrovascular accident” OR “ischemic stroke” OR poststroke) AND TITLE-ABS-KEY (“mental imagery” OR “kinesthetic imagery” OR “motor imagery” OR “visual imagery” OR “mental practice” OR “mental training” OR “mental rehearsal” OR visualiz* OR visualis* OR “internal verbali*” or “sensory imag*”)) AND TITLE-ABS-KEY (fim OR barthel OR ADL OR “activities of daily living” OR “daily life tasks” OR “activities of daily life” OR “quality of life” OR “patient satisfaction”)

### Selection of studies

Articles were eligible for inclusion if they met the following criteria: (1) randomized controlled trial design; (2) full-text manuscript published in English in a peer-reviewed journal; (3) stroke survivors at any stage of recovery as study participants; (4) an experimental group that received MI training in isolation or in combination with conventional therapy or in combination with another experimental therapy; (5) a comparison group that received conventional therapy or the other experimental therapy, either in isolation or paired with a placebo treatment; and (6) reported results on an outcome measure that assessed level of patient independence during the performance of three or more ADL. The requirement of three or more ADL is in line with previous systematic reviews (Janaudis-Ferreira et al., 2014).

### Identification of the relevant literature

Once duplicates were removed, five reviewers (KL, CH, JH, CM, and NB) screened each title and abstract for inclusion. The article was added to the full-text review if relevance was unclear from abstract screening. Each full-text article was then independently assessed for review inclusion by two of five reviewers (KL and NB). Disagreements regarding inclusion were resolved by a third reviewer (AL). Reference lists of the included studies were screened to identify additional relevant articles. Two reviewers (KL and NB) then independently extracted data from each study and organized the data into two separate tables, one for each reviewer, including the following elements: study population and setting, description of the intervention, target outcome measures, key results related to ADL independence, and study quality.

### Methodological quality assessment

We used the Physiotherapy Evidence Database (PEDro) scale to assess methodological quality of the included studies (Cashin and McAuley, 2020). The PEDro scale consists of eight questions that assess the internal validity of a study and two questions that assess the study’s statistical completeness (Cashin and McAuley, 2020). It defines the quality of a survey as excellent with a score of 9–10, good with a score of 6–8, fair with a score of 4–5, and poor with a score below 4. For complex interventions, such as those commonly administered by an occupational therapist, a total PEDro score of 8/10 is considered optimal as it is often impossible to blind participants or the therapists administering treatment (Cashin and McAuley, 2020). Two reviewers familiar with the PEDro (KL and NB) independently assessed the quality of each study, with any disagreement resolved by a third reviewer (AL).

### Data analysis and synthesis

The two extraction tables organized by KL and NB were then compared and the data synthesized to create a single data table. The effectiveness of MI was further analyzed by categorizing the studies according to modes of MI instruction. A traditional meta-analysis was not conducted due to the small number of studies within each category and the heterogeneity of measurements used in the studies. Instead, this review applied a narrative synthesis approach to review MI effectiveness and included a calculation of effect size (ES) on outcomes relevant to ADL independence in each of the studies. This approach is supported by the Cochrane Handbook, especially when heterogeneity in measurement is a concern (McKenzie and Brennan, 2020). The advantage of using ES to support narrative synthesis is that it allows the comparison of MI effects on a uniform scale to facilitate the quantification and interpretation of treatment effects. The ES was calculated by dividing the differences in mean scores post-intervention between the two comparison groups by the pooled baseline standard deviation; ESs of 0.8, 0.5, and 0.2 are considered large, medium, and small, respectively (Cohen, 2013).

## Results

**Figure 1.**
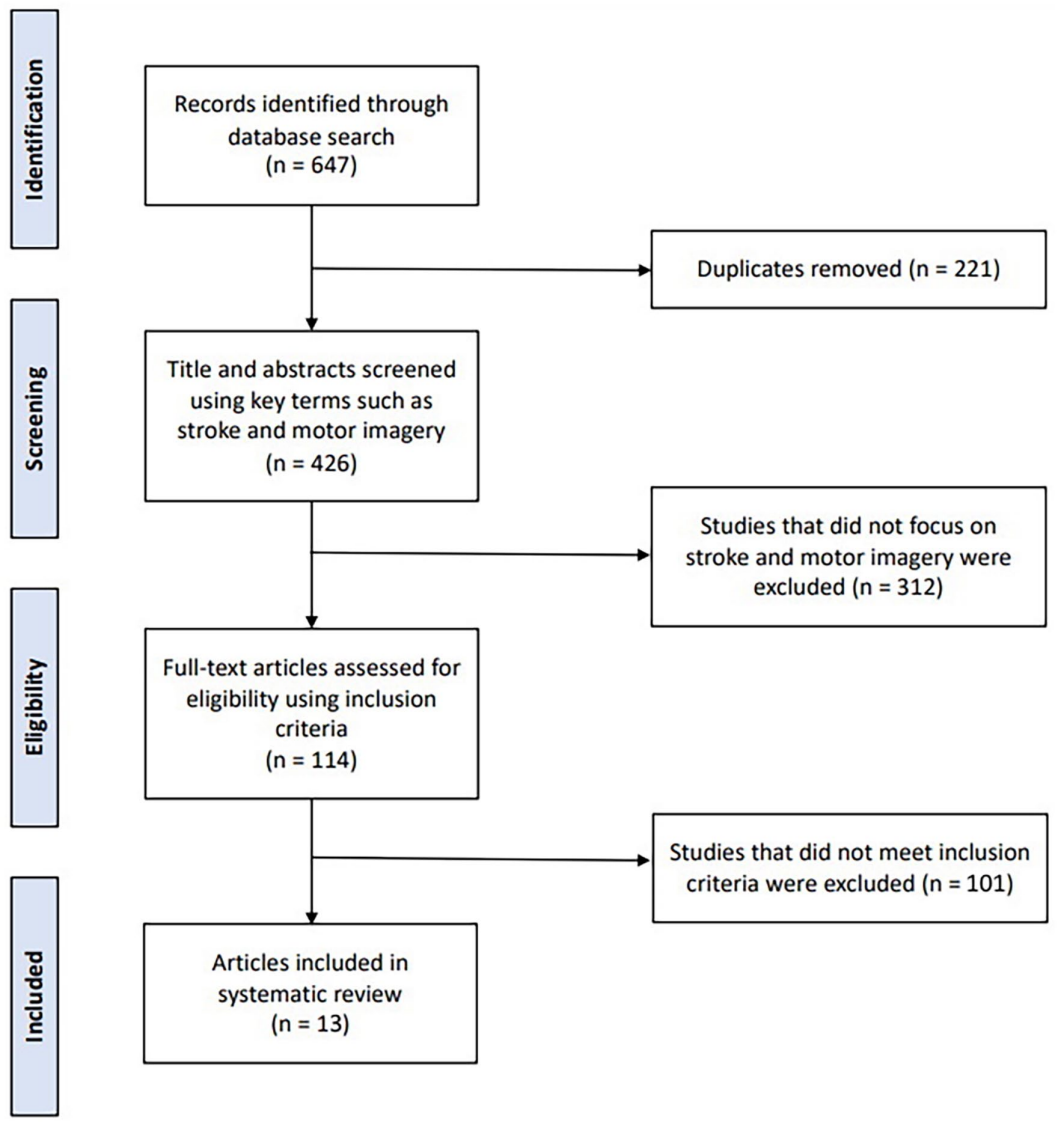
PRISMA flow diagram of the article screening process.

The search results are presented in the PRISMA flow diagram (Figure 1). The initial search yielded 647 titles and abstracts. After the removal of 221 duplicates, 426 titles and abstracts were screened, 114 articles were included full-text screening, and 101 articles were excluded as they did not meet inclusion criteria. A total of 13 articles met the inclusion criteria and are summarized in Table 2. Included studies were carried out in Hong Kong (Liu et al., 2004, 2009), the Netherlands (Braun et al., 2012), Scotland (Ietswaart et al., 2011), China (Pan et al., 2019; Yin et al., 2022), South Korea (Ji et al., 2021; Nam et al., 2019; Park, 2015, 2020; Park et al., 2015), and two in unspecified locations (Ahmad et al., 2014; Kulkarni and Sureshkumar, 2018).

**Table 2.** Descriptive summary and the Physiotherapy Evidence Database (PEDro) score of reviewed studies.

Authors	Sample	Intervention	Outcomes	ADL Outcome Results	Pedro
Ahmad et al (2014)	*N* = 40 EG1 (*N* = 10) Mean age: Not stated Mean time post-stroke: Not stated EG2 (*N* = 10) Mean age: Not stated Mean time post-stroke: Not stated EG3 (*N* = 10) Mean age: Not stated Mean time post-stroke: Not stated CG (*N* = 10) Mean age: Not stated Mean time post-stroke: Not stated	EG1: One 50-minute session of audio-cued motor imagery training EG2: One 50-minute session of visual-cued motor imagery training EG3: One 50-minute session of audio and visual-cued motor imagery training CG: One 50-minute session of task orienting training **Imagined actions:** Reaching for and drinking from a cup	**ADL** Barthel Index Other • Action Research Arm Test • Motor Activity Log	All groups improved on the BI following the treatment session, with no significant differences between groups (*p* = 0.98).	4
Braun et al. (2012)	*N* = 36 EG (*N* = 18; 9 F) Mean age: 77.9 years Mean time post-stroke: 4.8 weeks CG (*N* = 18; 13 F) Mean age: 77.7 years Mean time post-stroke: 6.1 weeks Participants were all inpatients at one of three nursing homes in the Netherlands.	EG: 6 weeks of multi-professional rehab in accordance with Dutch guidelines, embedded with 10–20 minutes of mental practice. CG: Did “homework” (physically practiced tasks they had difficulty with) **Imagined actions:** Two set tasks (walking, drinking), one personalized task	**ADL** Barthel Index **Other** • Nine Hole Peg Test • Goal Attainment Scaling • Berg Balance Scale • 10 Metre Walk Test • Motricity Index	No significant differences between groups on the BI at intervention conclusion (*p* = 0.463) or at 6 months follow up (*p* = 0.826).	7
Ietswaart et al. (2011)	*N* = 121 EG (*N* = 41; 18 F) Mean age: 69.3 years Mean time post-stroke: 82 days CG1 (*N* = 39; 17 F) Mean age: 68.6 years Mean time post-stroke: 90.8 days CG2 (*N* = 41) Mean age: 64.4 years Mean time post-stroke: 80.5 days Participants were a mix of outpatient and inpatients recruited from three hospitals in Scotland.	EG: 45-minute supervised motor imagery sessions 3x/week + 30-minute independent sessions 2x/week for 4 weeks CG1: 45-minute supervised visual imagery sessions 3x/week + 30-minute independent sessions 2x/week for 4 weeks CG2: 45-minute supervised conventional therapy sessions 3x/week + 30-minute independent sessions 2x/week for 4 weeks **Imagined actions:** Elementary and goal-directed upper extremity movements (e.g., reaching and grasping), ADL (e.g., buttoning a shirt), and mental rotation of hands	**ADL** Barthel Index **Other** • Action Research Arm Test • Modified Functional Limitation Profile	At intervention conclusion, all groups improved significantly on the BI (*p* = 0.019) with no differences between groups (*p* = 0.38).	7
Ji et al. (2021)	*N* = 42 EG (*N* = 21; 7 F) Mean age: 61.75 years Mean time post-stroke: 62.7 months CG (*N* = 21; 8 F) Mean age: 53.29 years Mean time post-stroke: 46.29 months	EG: 30 minutes home-based motor imagery for 8 weeks CGI: 30 minutes home-based upper extremity exercise program Both groups received conventional therapy. **Imagined actions:** Elementary and goal-directed upper extremity movements, mental rotation of hands	**ADL** Modified Barthel Index **Other** • Manual Function Test • Fugl-Meyer Assessment	At intervention conclusion, both groups improved significantly on the MBI (*p* < 0.05) with no differences between groups.	5
Kulkarni and Sureshkumar (2018)	*N* = 40 Group allocation and baseline characteristics not provided.	EG: 90-minute sessions of paired physical and mental practice of lower body exercises 6x/week for 6 weeks EG: 90-minute sessions of physical lower body exercises 6x/week for 6 weeks **Imagined actions:** Series of trunk control exercises	**ADL** Functional Independence Measure Other • Trunk Impairment Scale	At intervention conclusion, significant between-group differences on the FIM (*p* < 0.0001).	3
Liu et al. (2004)	*N* = 26 EG (*N* = 26; 15 F) Mean age: 71 years Mean time post-stroke: 12.3 days CG (*N* = 20; 9 F) Mean age: 72.7 years Mean time post-stroke: 15.4 days Participants were all inpatients on a stroke rehabilitation unit in Hong Kong.	Each group completed 15 treatment sessions (1 hour/day, 5 days/week, for 3 weeks) of progressive difficulty EG: Task analysis techniques (week 1); problem identification (week 2); videotaped actual task performance with self-evaluation and self-proposed solutions (week 3) CG: Conventional occupational therapy **Imagined actions:** Fifteen different ADL primarily related to household management (e.g., preparing fruit, making the bed, counting change)	**ADL** 7-point Likert scale rating of ADL task performance on 15 trained and 5 untrained daily tasks Other • Fugl-Meyer Assessment • Color Trails Test	EG performed better on trained and untrained ADL tasks (*p* < 0.001) immediately following the intervention, and the trained ADL tasks at 1 month follow up (*p* < 0.001).	7
Liu et al. (2009)	*N* = 33 EG (*N* = 16; 8 F) Mean age: 70.8 years Mean time post-stroke: 12.2 days CG (*N* = 17; 5 F) Mean age: 69.7 years Mean time post-stroke: 12.3 days Participants were all inpatients at a rehabilitation hospital in Hong Kong.	Each group completed 15 treatment sessions (1 hour/day, 5 days/week, for 3 weeks) of progressive difficulty EG: Task analysis techniques (week 1); problem identification (week 2); videotaped actual task performance with self-evaluation and self-proposed solutions (week 3) CG: Conventional occupational therapy **Imagined actions:** Fifteen different ADL primarily related to household management (e.g., preparing fruit, making the bed, counting change)	**ADL** 7-point Likert scale rating of ADL task performance on five trained and five untrained daily tasks	At intervention conclusion, EG group improved on four of five (*p* = 0.001–0.026) trained tasks and three of five untrained tasks (*p* = 0.025–0.049). CG improved on one trained task (*p* = 0.021) and two untrained tasks (*p* = 0.042–0.045).	6
Nam et al. (2019)	*N* = 20 EG (*N* = 10; 4 F) Mean age: 61.8 years Mean time post-stroke: 67.4 days CG (*N* = 10; 6 F) Mean age: 59.6 years Mean time post-stroke: 74 days Participants were all inpatients admitted to the Department of Rehabilitation of a hospital in Korea.	EG: Twenty minutes motor imagery training with videotaped guidance following conventional therapy five times/week for 4 weeks. Both groups received 30-minute conventional therapy five times/week for 4 weeks. **Imagined actions:** Four items from the Manual Function Test (grasp, pinch, carry a cub, peg-board) and two items from the Stroke Upper Limb Capacity Scale (slide object across table, hold a drinking glass)	**ADL** Functional Independence Measure Other • Fugl-Meyer Assessment • Manual Function Test	At intervention conclusion, both groups improved significantly on the FIM (*p* < 0.05) with no differences between groups (*p* = 0.481).	6
Pan et al. (2019)	*N* = 42 EG (*N* = 21; 5 F) Mean age: 63.38 years Mean time post-stroke: 4.96 months CG (*N* = 21; 9 F) Mean age: 64.14 years Mean time post-stroke: 5.13 months 21 experimental, 21 control Participants were all inpatients at a rehabilitation hospital in China.	Both groups received 10 30 minutes transcranial magnetic stimulation (TMS) sessions at 1 Hz over 4 weeks EG: TMS + 30 minutes of listening to audiotaped motor imagery script CG: TMS + 30 minutes of listening to audiotaped relaxation script **Imagined actions:** A combination of elementary upper extremity movements and ADL (e.g., open a door, fold a piece of paper, writing)	**ADL** Modified Barthel Index **Other:** • Box and Block Test • Wolf Motor Function Test • Fugl-Meyer Assessment	At intervention conclusion, both groups improved on MBI (*p* < 0.01), with significant between-group differences favoring EG (*p* < 0.001).	7
Park (2015)	*N* = 26 EG (*N* = 13; 6 F) Mean age: 60.9 years Mean time post-stroke: 15.9 months CG (*N* = 13; 4 F) Mean age: 63.1 years Mean time post-stroke: 14.4 months Participants recruited through a rehabilitation hospital in Korea.	Both groups received modified constraint-induced movement therapy 5 days/week for 6 weeks (15 hours practice, 120 hours restraint) EG: Additional 30 minutes of listening to audiotaped motor imagery script following treatment sessions **Imagined actions:** Reaching and grasping an object, using a pencil or pen, and turning a page in a book.	**ADL** Modified Barthel Index **Other:** • Action Research Arm Test • Fugl-Meyer Assessment	At intervention conclusion, both groups improved on MBI (*p* < 0.05), with significant between-group differences favoring EG (*p* < 0.05).	6
Park et al. (2015)	*N* = 29 EG (*N* = 14; 4 F) Mean age: 60 years Mean time post-stroke: 18 months CG (*N* = 15; 4 F) Mean age: 58 years Mean time post-stroke: 16 months Participants were receiving treatment for hemiplegia at a hospital in Korea.	EG: 20 minutes of conventional occupational therapy + 10 minutes of listening to audiotaped motor imagery script 5 days/week for 2 weeks. CG: 30 minutes conventional occupational therapy 5 days/week for 2 weeks. **Imagined actions:** Stacking plastic cups, turning a page, and handling beans.	**ADL** Modified Barthel Index **Other:** • Action Research Arm Test • Fugl-Meyer Assessment	At intervention conclusion, EG improved significantly more than CG (*p* < 0.05) on the MBI.	4
Park (2020)	*N* = 68 EG (*N* = 24; 18 F) Mean age: 66.8 years Mean time post-stroke: 22.79 months CG (*N* = 24; 18 F) Mean age: 65.79 years Mean time post-stroke: 21.71 months Participants were all recruited through a rehabilitation hospital in Korea.	Both groups received 30 minutes of electromyogram-triggered neuromuscular electrical stimulation EG: NMES with computer guided motor imagery of vigorous movements with affected upper limb **Imagined actions:** Sport-related movements involving the upper limb (e.g., spiking a volleyball, throwing a baseball)	**ADL** Modified Barthel Index **Other:** • Action Research Arm Test • Fugl-Meyer Assessment	At intervention conclusion, both groups improved significantly on the MBI (*p* < 0.05). There were no between-group differences.	8
Yin et al. (2022)	*N* = 32 EG (*N* = 16; 3 F) Mean age: 56.9 years Mean time post-stroke: 52.3 days CG (*N* = 16; 4 F) Mean age: 57.1 years Mean time post-stroke: 58.1 days Participants were all neurology inpatients at a hospital in China.	**Intervention** Both groups received therapeutic exercise and conventional occupational therapy 5x/week, 3-hour sessions. EG: Additional 20 minutes of listening to audiotaped motor imagery script following treatment sessions. **Imagined actions:** Bed transfer, sit to stand transfer, weight shifting in standing position, walking and using the stairs.	**ADL** Functional Independence Measure **Other** • Fugl-Meyer Assessment • Berg Balance Scale	At intervention conclusion, EG improved significantly more than CG (*p* < 0.001) on the FIM.	6

ADL: activities of daily living; BI: Barthel Index; CG: control group; EG: experimental group; FIM: Functional Independence Measure; MBI: Modified Barthel Index; NMES: neuromuscular triggered electrical stimulation.

In terms of quality, the PEDro score indicated that one study was rated poor, three rated fair, and nine rated good. A summary of the articles and their PEDro scores were presented in Table 2, and details of the quality of evidence were presented in Table 3. The most common sources of bias were lack of blinding (*n* = 13), intention-to-treat analysis (*n* = 10), and concealed allocation (*n* = 7).

**Table 3.** Quality of evidence assessed by the Physiotherapy Evidence Database (PEDro) scale for individual studies.

Trial	Random allocation	Concealed allocation	Baseline comparability	Blind participants	Blind therapists	Blind assessors	<15% dropouts	Intention-to-treat analysis	Between-group differences reported	Point estimate and variability reported
Ahmad et al. (2014)	X					X			X	X
Braun et al. (2012)	X	X	X			X		X	X	X
Ietswaart et al. (2011)	X	X				X	X	X	X	X
Ji et al. (2021)	X		X				X		X	X
Kulkarni and Sureshkumar (2018)	X								X	X
Liu et al. (2004)	X	X	X			X	X		X	X
Liu et al. (2009)	X	X	X			X	X		X	
Nam et al. (2019)	X		X			X			X	X
Pan et al. (2019)	X		X			X	X	X	X	X
Park (2015)	X		X			X	X		X	X
Park et al. (2015)	X		X						X	X
Park (2020)	X	X	X			X	X	X	X	X
Yin et al. (2022)	X	X	X			X			X	X

The studies included a total of 555 participants. The average time since the participants experienced their stroke ranged from 2 weeks in Liu et al. (2004) to over 5 years in Ji et al. (2021). Imagined actions varied in complexity, with some studies involving imagery of elementary movements, such as opening and closing a fist (Nam et al., 2019), while others had participants imagine completing household management activities (Liu et al., 2004, 2009). Interventions ranged from a single session (Ahmad et al., 2014) to five sessions per week for 8 weeks (Ji et al., 2021). The cumulative dose of MI training ranged from 50 minutes (Ahmad et al., 2014) to 1680 minutes in Ji et al. (2021).

Three studies used the BI to measure independence in BADL (Ahmad et al., 2014; Braun et al., 2012; Ietswaart et al., 2011), while the Modified Barthel Index (MBI) was used in five studies (Ji et al., 2021; Pan et al., 2019; Park, 2015, 2020; Park et al., 2015). Independence in ADL was measured in two studies using the full FIM (Kulkarni and Sureshkumar, 2018; Nam et al., 2019). Yin et al. (2022) used the transfer and locomotion subscales of the FIM as opposed to the scale in its entirety. An original outcome measure was employed in two studies (Liu et al., 2004, 2009). In this measure, participants were observed performing different IADL, with the level of support needed for them to successfully perform the task rated on a 7-point Likert Scale.

**Figure 2.**
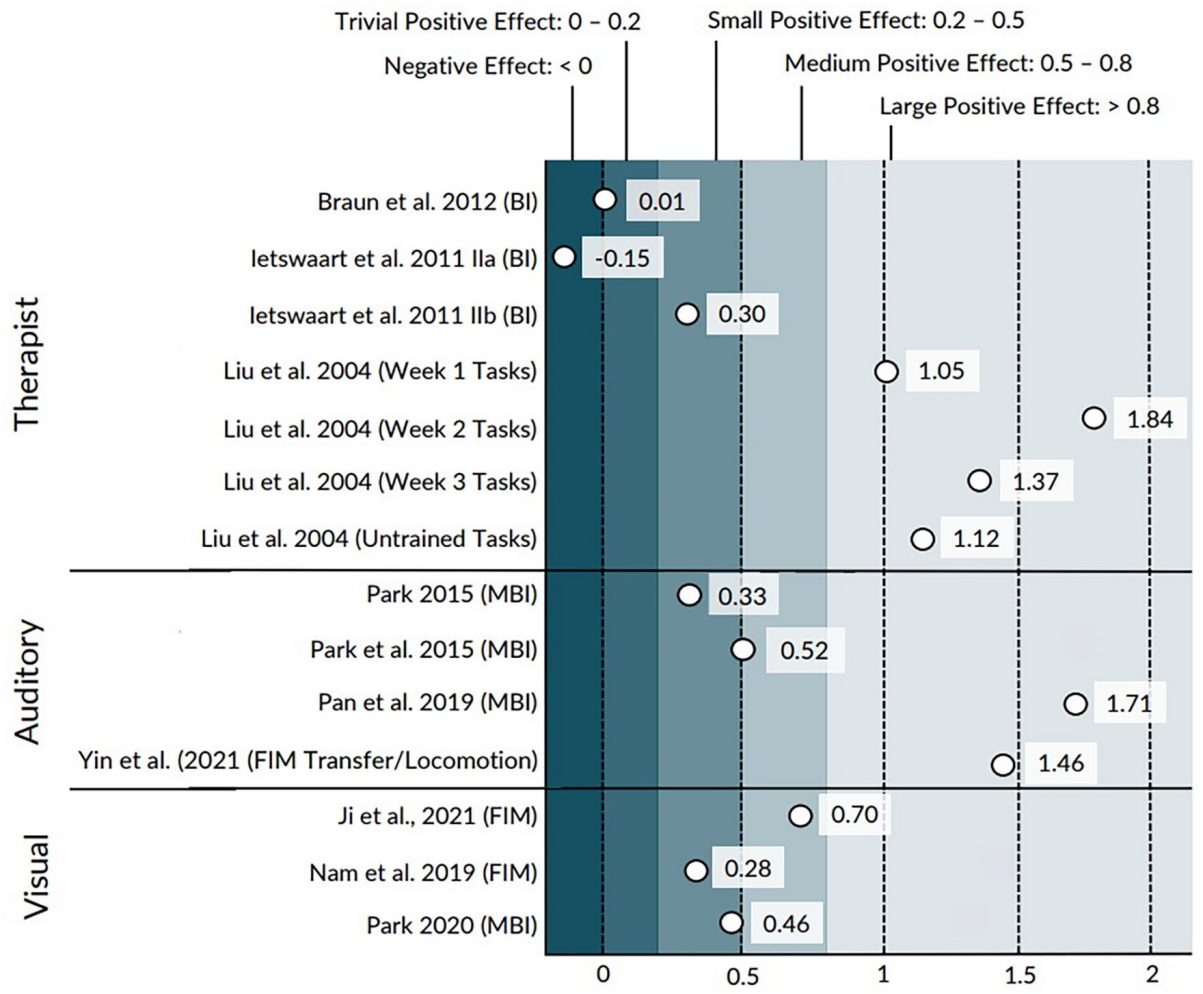
Effect sizes for each study, separated by mode of training delivery. Outcome measures are displayed in parentheses. IIa: control group A; IIb: control group B; BI: Barthel Index; MBI: Modified Barthel Index; FIM: Functional Independence Measure.

The primary mode of MI delivery included direct therapist instruction (Braun et al., 2012; Ietswaart et al., 2011; Liu et al., 2004, 2009), pre-recorded audio scripts (Pan et al., 2019; Park, 2015; Park et al., 2015; Yin et al., 2022), video recordings (Nam et al., 2019), text instructions presented on a computer screen (Park, 2020), and images shown on a computer screen (Ji et al., 2021). These studies were categorized into modes of MI instruction for further analysis, thus leading to the generation of three categories: therapist-directed MI (*n* = 4), audio-guided MI (*n* = 4), and visual-guided MI (*n* = 3). The calculated ESs of studies are available in Figure 2. ESs were calculated from the original BI, 2 out of 10 studies; the MBI, 4 out of 10 studies; the FIM, 3 out of 10 studies; and an original task observation assessment, 1 out of 10 studies. Two fair-quality studies did not provide any information on the mode of MI instruction (Ahmad et al., 2014; Kulkarni and Sureshkumar, 2018). These two studies were therefore not categorized, and their ESs were not calculated. The ES of one study in the therapist-directed MI category was not calculated as the manuscript did not contain measures of variability for the target outcome measure (Liu et al., 2009). Below are the findings organized according to the mode of MI instruction.

### Therapist-directed imagery

Four studies, with a total of 245 participants, delivered MI training primarily through in-person therapist instruction (Braun et al., 2012; Ietswaart et al., 2011; Liu et al., 2004, 2009). Three of the studies paired MI training with physical task practice (Braun et al., 2012; Liu et al., 2004, 2009), and one delivered MI training in isolation (Ietswaart et al., 2011). All studies were considered good quality. Three of the studies received a PEDro score of 7 (Braun et al., 2012; Ietswaart et al., 2011; Liu et al., 2004), and one received a PEDro score of 6 (Liu et al., 2009). Participants were in the sub-acute stage of stroke recovery in all studies. The ESs ranged from −0.15 to 1.84, which spans from a trivial effect in favor of the control group to a large effect in favor of the experimental group (Figure 2).

Ietswaart et al. (2011) examined the effect of a MI training program on a mix of inpatients and outpatients. Therapists with a psychology or occupational therapy background delivered highly structured 45-minute sessions broken down into three components: imagery of elementary and goal-directed movements, MI guided by a mirror or video, and mental rotation of hands. Participants additionally completed two sessions of independent MI training that were guided by 30-minute audio recordings. The two control groups received either dose-matched routine care or imagery training that focused on the visualization of objects, as opposed to imagery of movements (Ietswaart et al., 2011). All groups improved similarly on the BI (Ietswaart et al., 2011).

Liu et al. (2004) and Liu et al. (2009) administered the same 3-week MI intervention. This program involved 1 hour of training each weekday on 1 of 15 household management tasks (e.g., making the bed, taking medication, washing dishes) for a total of five trained tasks per week. Working directly with an occupational therapist, participants practiced breaking down each task and identifying deficits in task performance, followed by the mental and then physical rehearsal of the task. In both studies, participants in the experimental group improved significantly more than controls (dose-matched conventional occupational therapy) on the support needed to complete both trained and untrained tasks (Liu et al., 2004, 2009). In Liu et al. (2004), these between-group differences tasks were maintained at 1 month follow up.

Braun et al. (2012) embedded MI into conventional therapy for stroke patients receiving rehabilitation at three different nursing homes. While therapists received protocol training, they were also encouraged to adapt training to the needs of each participant. Participants were trained on two set tasks (e.g., walking, drinking) and a third task of their choosing. While participants were provided with an imagery log to track independent practice, unclear participant reporting limited its interpretation. No significant differences were documented by researchers between the experimental group and controls (dose-matched usual care) on the BI, either at study conclusion or 6 month follow up (Braun et al., 2012).

When considered together, these four good-quality studies provide mixed evidence for the benefits of therapist-delivered MI training on ADL independence. The null results from Braun et al. (2012) and Ietswaart et al. (2011) (*n* = 157) suggest that this training does not have a significant effect on the BI, a measure of BADL independence. However, the statistically significant effects documented by Liu et al. (2004) and Liu et al. (2009) suggest that therapist-delivered training may be effective intervention if used to target independence in IADL. However, these results were drawn from a comparatively smaller sample size (*n* = 59) and involved the use of a non-validated measure to assess ADL independence.

### Non-therapist directed

#### Audio-guided MI

Four studies, with a total of 130 participants, delivered MI training through pre-recorded audio scripts (Pan et al., 2019; Park, 2015; Park et al., 2015; Yin et al., 2022). MI training in all studies was standardized across participants (Pan et al., 2019; Park, 2015; Park et al., 2015; Yin et al., 2022). All studies included relaxation exercises at the start of each session and emphasized MI of ADL tasks using the affected limb (Pan et al., 2019; Park, 2015; Park et al., 2015; Yin et al., 2022). These studies were considered fair to good quality. The PEDro scores of the included studies were 4 (Park et al., 2015), 6 (Park, 2015), 6 (Yin et al., 2022), and 7 (Pan et al., 2019). The ESs ranged from 0.33 to 1.71, which spans a small to large effect in favor of the experimental group (Figure 2).

Two studies paired MI training with conventional occupational therapy (Park et al., 2015; Yin et al., 2022). In Park et al. (2015), patients more than 6 months post-stroke received 20 minutes of occupational therapy followed by 10 minutes of MI guided by audio recordings. Controls received dose-matched occupational therapy. The experimental group improved significantly more than controls on the Korean MBI. Yin et al. (2022) investigated MI training in hospitalized patients more than 1 month post-stroke. Participants completed 20 minutes of MI training in addition to occupational therapy, while controls received occupational therapy in isolation. The experimental group improved significantly more on the FIM at study conclusion than controls, who received occupational therapy in isolation (Yin et al., 2022).

Two studies paired MI training with treatment other than conventional therapy (Pan et al., 2019; Park, 2015). Park (2015) examined the effect of MI training paired with modified constraint-induced movement therapy (mCIMT) in patients more than 6 months post-stroke. Participants in both groups received 30 minutes of therapy five times per week. The experimental group additionally listened to 30 minute pre-recorded MI scripts directly following treatment sessions. The researchers recorded significantly greater gains on the Korean MBI in the experimental group than controls, who received mCIMT in isolation (Park, 2020). In Pan et al. (2019), hospitalized patients between 3 and 12 months post-stroke listened to pre-recorded MI scripts for 30 minutes during repetitive transcranial magnetic stimulation (rTMS). Controls listened to pre-recorded relaxation scripts while receiving rTMS. Both groups received dose-matched conventional therapy outside of sessions. The experimental group exhibited significantly greater improvements on the MBI than the controls (Pan et al., 2019).

The three good-quality studies (Pan et al., 2019; Park, 2015; Yin et al., 2022) in this group both recorded significant differences between groups, with large effects favoring the experimental group. These results suggest that MI training delivered through audio recordings improves independence in ADL when added to another intervention. However, the findings must be interpreted with caution as two studies did not control for the additional therapeutic time spent listening to the pre-recorded MI scripts (Park, 2015; Yin et al., 2022). The remaining fair-quality study (Park et al., 2015) provides some additional support for the benefits of audio-delivered MI training, although ESs were small to moderate.

#### Visual-guided imagery

Three studies, with a total of 130 participants delivered MI training that was primarily guided through visual information (Ji et al., 2021; Nam et al., 2019; Park, 2020). The types of visual input included videos (Nam et al., 2019), text displayed on a computer screen (Park, 2020), and photographs (Ji et al., 2021). The included studies were considered fair to good quality, with PEDro scores of 5 (Ji et al., 2021), 6 (Nam et al., 2019), and 8 (Park, 2020). The ESs ranged from 0.28 to 0.70, which spans a small to medium effect in favor of the experimental group (Figure 2).

In Park (2020), participants more than 6 months post-stroke practiced MI for 30 minutes while receiving neuromuscular triggered electrical stimulation (NMES) to the forearm of the hemiplegic upper limb. The MI training was guided by written text on a computer screen and involved imagery of sports-related upper extremity movement (e.g., throwing a baseball and swinging a tennis racket). Controls received the same amount of NMES in isolation. Both the experimental and control groups improved significantly on the MBI, with no significant differences between groups.

Ji et al. (2021) adapted a graded MI training program from complex regional pain syndrome protocols for patients with chronic stroke. The home-based program was delivered daily through a smartphone over 8 weeks. Participation was limited to individuals more than 3 months post-stroke. Each 30-minute session included three components: mental rotation of hands, MI of upper limb movements guided by photographs, and mirror therapy (Ji et al., 2021). Dose-matched physical practice of the imagined tasks served as the control intervention. While no significant differences between groups were recorded, ES calculations indicated a moderate effect on the FIM in favor of the experimental group. This study received a PEDro score of 5, indicating fair quality.

Nam et al. (2019) also delivered MI training through a smartphone. Participants in the early sub-acute phase of stroke were guided through imagery by a reversed video of themselves performing the target movement with their unaffected limb, thus creating the appearance of using their affected limb during the movement. The imagined actions were basic activities taken from two post-stroke upper limb assessments (Manual Function Test, Stroke Upper Limb Capacity Scale). Participants completed the 30-minute imagery sessions following conventional therapy, which the controls received in isolation. Results indicated no significant between-group differences on the MBI

None of the three studies recorded significant differences between the experimental and control groups on measures of ADL independence. This lack of significance in two studies of good methodological quality, with supporting evidence from one fair-quality study, suggests that MI training is not an effective intervention when delivered indirectly through a visual mode of instruction if used to target independence in ADL post-stroke.

## Discussion

To our knowledge, this is the first systematic review to examine the effect of MI training post-stroke using independence in ADL as a primary outcome. The included studies used several approaches to deliver MI training, with varying results. While all studies documented the benefits of MI training on the level of assistance needed to perform ADL, only half recorded significant differences between experimental and control groups.

Between-group differences occurred in all interventions that added audio-delivered MI training to other treatments (conventional occupational therapy, TMS, mCIMT). However, these differences must be interpreted with caution. The total number of participants was relatively small, and the range of documented ESs was large (0.33–1.71). Additionally, it is not possible to rule out the placebo effect in two of the studies. While more high quality research is needed in the area, the documented differences remain promising for stroke rehabilitation. Stroke patients commonly report a desire to receive rehabilitation of higher intensity and duration than currently provided in standard care (Luker et al., 2015). Inpatients frequently perceive a lack of practice opportunities outside of supervised treatment sessions (Luker et al., 2015). MI training using audio-guided scripts offers a low risk means by which patients can engage in additional therapy without relying on the presence of others.

The present review found no evidence supporting the use of visual-guided MI training delivery to facilitate ADL independence, while evidence for therapist-directed MI training was mixed. There are a few possible reasons for this mixed evidence. The two studies that recorded between-group differences following therapist-directed training contained several unique characteristics (Liu et al., 2004, 2009). Both involved using MI as a cognitive strategy to identify and problem-solve challenges related to task performance. Liu et al. (2004) and Liu et al. (2009) were also the only studies to measure independence in IADL, and did so using a non-validated measure. Finally, participants were on average around 2 weeks post-stroke, earlier than other studies using therapist-directed training (Braun et al., 2012; Ietswaart et al., 2011).

MI training is proposed to be particularly valuable in the early stages of stroke recovery when physical function of the affected limbs is especially impaired (Sharma et al., 2006). Barclay et al. (2020) documented no relationship between the stage of stroke recovery and the effect of MI training on upper extremity outcomes. In the present review, the average time post-stroke in studies that delivered MI training through audio recordings to positive results ranged from <2 to 18 months. It is thus unclear from this review if the stage of stroke recovery impacts the effect of MI training on ADL performance, or if this effect is impacted by the mode of training instruction.

Another intervention characteristic of interest is training dosage. Any assessment of a dose-response relationship is limited as we were unable to determine the cumulative MI training dose of several interventions (Braun et al., 2012; Liu et al., 2004, 2009). While all studies that delivered MI training through audio recordings reported benefits of ADL performance, the cumulative dose of training ranged from 100 to 900 minutes. Recent reviews have documented no clear relationship between the dose of MI training and its effect on upper extremity function following stroke (Barclay et al., 2020; Gaughan and Boe, 2022). The absence of a clear relationship between MI training effectiveness and training dosage documented here is therefore in line with previous research.

All included studies aside from Liu et al. (2004) and Liu et al. (2009) measured ADL independence using a variation of either the BI or the FIM. The BI and the FIM are validated measures that involve rating the level of assistance an individual requires to perform different activities. The two measures have comparable psychometric properties for stroke patients (Schepers et al., 2006). The MBI was developed to address the perceived insensitivity of the BI (Quinn et al., 2011). While the original BI uses a two- or three-step scale to rate independence, a five-step scale is used for rating all tasks in its modified version. The MBI has been recommended for use over the original index in future stroke trials (Quinn et al., 2011).

Occupational therapy was directly mentioned in most studies. All four studies that delivered MI training through a healthcare professional involved occupational therapists in training delivery. Several studies paired MI training with conventional occupational therapy, including three studies that used audio recordings as their mode of instruction. Several other studies paired training with general conventional therapy that likely involved occupational therapy or interventions within the profession’s scope, such as CIMT. This prominence of occupational therapy within the reviewed literature highlights the potential relevance of MI training to the profession’s established role in stroke rehabilitation.

A key component of occupational therapy’s role is enabling patient participation in meaningful activities (Steultjens et al., 2003). As ADL are viewed by many stroke survivors as meaningful, independence in ADL is a relevant outcome measure for occupational therapists (Eriksson and Tham, 2010). For some stroke survivors, independence in ADL is valuable because it facilitates participation in other meaningful activities related to work, leisure, or social-related roles (Wood et al., 2010). Other survivors may view ADL as meaningful in and of themselves, with increased independence in these activities providing a sense of autonomy (Wood et al., 2010).

This review indicates the need for greater methodological rigor in studies that investigate MI training as a treatment for ADL independence. Blinding of participants and therapists is often challenging to accomplish in rehabilitation trials (Santaguida et al., 2012). However, challenges in meeting such criteria renders the blinding of assessors especially important to the study’s internal validity. While comprehensive blinding is difficult to accomplish in rehabilitation trials, concealed allocation and intention-to-treat analysis are comparatively straightforward to implement. Concealed allocation, which prevents selection and confounding biases, was not discussed in 8 of the 13 studies. This result is in line with previous findings that concealed allocation commonly goes unreported in stroke rehabilitation (Santaguida et al., 2012). Intention-to-treat analysis is not required when all participants receive the treatment as assigned. Providing information on treatment compliance, and statistical measures taken if such compliance is lacking, is essential to confidence in the reported results.

### Limitations and future studies

The current review has several limitations. While the included studies come from a diverse range of countries, we did restrict articles to those published in English. This restriction was applied at the start of the literature search, and therefore we cannot be certain how many articles were excluded that otherwise met the review’s eligibility criteria. Second, our definition of an ADL outcome measure was reached through a joint discussion of three practicing occupational therapists and consultation with the literature. However, some may argue that our chosen definition is overly conservative and led to the unnecessary exclusion of relevant articles. For example, several excluded studies used the Motor Activity Log (MAL) as an outcome measure. In the MAL, participants post-stroke rate the use of their affected arm in the context of ADL along two scales: amount of use and quality of movement (van der Lee et al., 2004). While ADL serve as the assessment context, neither scale specifically focuses on participant independence in the target activities. We thus excluded the MAL as an outcome measure. The high heterogeneity of the included studies limits our ability to determine critical factors of successful MI training interventions. Finally, the categorization of studies based on modes of MI instruction provided additional value to this review but led to the exclusion of two lower-quality studies in the interpretation of findings. These two studies met inclusion criteria but lacked essential information about their respective training programs.

Future studies that use audio-guided training should consider incorporating a placebo treatment such as pre-recorded relaxation scripts into the control intervention to increase confidence in the effects of MI training. We also recommend that future studies include replicable MI training protocols to facilitate comparison between otherwise similarly described studies.

## Conclusion

This review examined the effect of MI training on ADL independence following stroke. Audio-based MI training consistently increased independence in ADL when paired with other rehabilitation methods. The lack of supervision required for such training could provide patients with additional therapeutic activity outside supervised treatment sessions. Findings were inconsistent for MI training delivered directly by a therapist. Treatment dosage and stage of stroke recovery did not affect training effectiveness. The small number of participants included in most reviewed studies indicates a need for further research into MI training’s effectiveness in the post-stroke population. We hope that the potential benefits documented in this review, together with methodological considerations for improving the quality of evidence, will encourage further development of MI training protocols for implementation in occupational therapy practice.

**Key findings**

- MI training delivered through audio recordings appears to facilitate ADL independence
- High heterogeneity in interventions limits the ability to draw conclusions on effective treatment characteristics.

**What the study has added**

We examined the effects of MI training on ADL post-stroke. Findings support audio-based training as an effective adjutant tool, with more research required for therapist-delivered training.
